# A Comparison of Sexual Outcomes in Primiparous Women Experiencing Vaginal and Caesarean Births

**DOI:** 10.4103/0970-0218.51237

**Published:** 2009-04

**Authors:** M Khajehei, S Ziyadlou, Rad M Safari, HR Tabatabaee, F Kashefi

**Affiliations:** Department of Midwifery, Fatemeh (P.B.U.H.) College of Nursing and Midwifery, Shiraz University of Medical Sciences, P.O. Box: 71645 - 111, Shiraz, Iran; 1 Department of Midwifery, Epidemiology Department Health and Nutrition Faculty, Shiraz University of Medical Sciences, P.O. Box: 71645 - 111, Shiraz, Iran

**Keywords:** Postpartum, caesarean section, normal vaginal delivery, sexual problems

## Abstract

**Background and Objective:**

We conducted this study to evaluate and compare postpartum sexual functioning after vaginal and caesarean births.

**Materials and Methods:**

This was a cross-sectional study that was carried out in postnatal health care in a hospital. A total of 50 primiprous women who had given birth 6-12 months ago and came to the hospital for postnatal care were asked to join the study. Forty of the women completed the entire questionnaire. Among these women, 20 delivered spontaneously with mediolateral episiotomy and 20 had elective caesarean section. Sexual function was evaluated by a validated, self-created questionnaire. A statistical evaluation was carried out by SPSS v.11. A two-part self-created validated questionnaire for data collection was administered regarding sexual function prior to pregnancy and 6-12 months postpartum.

**Results:**

The median time to restart intercourse in the normal vaginal delivery with episiotomy (NVD/epi) group was 40 days and in the caesarean section (C/S) group was 10 days postpartum. The most common problems in the NVD/epi group was decreased libido (80%), sexual dissatisfaction (65%), and vaginal looseness (55%). In the C/S group, the most common problems were vaginal dryness (85%), sexual dissatisfaction (60%), and decreased libido (35%). There were clinically significant differences between the two groups regarding sexual outcomes, but these differences were not statically significant.

**Conclusion:**

Postnatal sexual problems were very common after both NVD/epi and C/S. Because sexual problems are so prevalent during the postpartum period, clinicians should draw more attention to the women's sexual life and try to improve their quality of life after delivery.

## Introduction

Traditionally, the postpartum period has been defined as beginning 1 hour after delivery of the placenta and lasting 6 weeks, at which time the uterus has regained its pre-pregnant size.(1) In a wider perspective, postpartum health will be considered in the following time period: immediate postpartum (birth to 6 months), short-term (3 to 6 months), and long-term (>6 months).(2,3)

During these times, normal activities will be resumed. Sexual activity may be resumed when the perineum is comfortable and when bleeding has diminished. The desire and willingness to resume sexual activity in the puerperium varies greatly among women, depending on the site status of healing of the perineal or vaginal incision and lacerations, the amount of vaginal atrophy secondary to breast-feeding, and return of libido.(4)

Although the median time to resume intercourse after delivery is 6 to 7 weeks, approximately one half of women who do so have dyspareunia. In a substantial number, dyspareunia lasts for 1 year or more [Table 1].(5,6) According to different studies, perineal morbidity, postpartum sexual problems, and other complaints are related to the method of delivery [Table 2]. Sexual problems are associated more with instrumental birth and episiotomy than caesarean section (C/S) or normal vaginal delivery (NVD).(7)

**Table 1:** Postpartum symptoms and complaints over time*

Symptom/Complaints	0-3 months (%)	3-6 months (%)	Over 6 months (%)
Presence of some health	94	81	31
problems (3)			
Perineal pain (23,18,11)	25-30	11	21
Sexual problem/dyspareuni (23,11)	19-35	26	11-49
Urinary stress incontinence (13)	8-34	10	3-7
Backache (11,9)	14-40	43	8-64
Hemorrhoid (11,12,23)	8-24	24	16
Fecal incontinence/bowel	9	12	1-3
problems (12,23)		
Extreme tiredness (9,11,23)	12-50	69	6-48
Depression (9,11,12,23)	7-30	19	4-20

* All methods of delivery are included.

**Table 2:** Postpartum symptoms and complaints relative to method of delivery

Symptom/Complaint	Time frame of report of symptoms	Spontaneous vaginal delivery%	Assisted vaginal delivery %	Caesarean section %
Presence of health problem (2)	8 weeks PP	84	96	93
Perinael pain (2)	8 weeks PP	7	30	-
Sexual problems (14)	6-7 months PP	23	39	27
Dyspareunia (11)	2^nd^ year PP	21	37	-
Urinary stress incontinence (25,26)	3 months PP	24	27	5
	6-7 months PP	11	18	2-7
Hemorrhoids (14)	6-7 months PP	25	6	11-16
Fecal incontinence bowel problems (27)	3 months PP	9	10-13	7
	6-7 months PP	11	19	12
Depression (14)	6-7 months PP	18	21	23
Caesarean wound pain (24)	3 months PP	N/A	N/A	10
	6-7 months PP	N/A	N/A	60

PP = Postpartum

Dyspareunia has also been observed in women who breastfeed their infants, suggesting that the lack of estrogen effect on the vagina is a cause of postpartum dyspareunia.(8)

What is the most disturbing in the literature on postpartum health is not the presence of widespread morbidity but the profound silence that surrounds this pivotal period in women's lives. Several studies noted that many women (up to 25%) with postpartum health problems did not consult a health professional.(2,9)

Although postpartum women have a responsibility to inform their health care providers, the burden of responsibility remains with the clinicians, midwives, and nurses. By means of some approach in order to promote sexual health, health care providers can help patients regain a sense of normalcy after childbirth.(10) The goal of this study was to evaluate sexual dysfunction after vaginal delivery compared with caesarean section.

## Materials and Methods

A total of 50 primiparous postpartum women between the ages of 17 and 40, who had presented in the postnatal health care clinic in the hospital, were asked to join this study during 2007. Inclusion criteria were: giving birth 6 months prior up to 1 year by means of caesarean section or vaginal delivery with episiotomy, negative history of instrumental vaginal delivery, and not having vaginitis. Informed consent was obtained through the provision of an information leaflet coupled with verbal reassurance that participation was entirely voluntary and that the participant could withdraw at any time. All of the participants were assured of confidentially and anonymity. In addition, all of the participants were supposed to put their answered questionnaires in a covered box.

A two-part, self-created questionnaire for data collection was administered regarding sexual functioning prior to pregnancy and 6-12 months postpartum. The first part assessed demographic and socio-economic status (age, level of education, employment, and partner's age, job, and level of education), obstetric history (type of birth, episiotomy, parity), and gynecological status (symptoms of vaginitis, breastfeeding). The second part of the questionnaire was completed concerning sexual functioning prior to pregnancy and at 6 months postpartum or more. This part consisted of 10 questions regarding problems in their sexual life. A total of 9 questions were multiple choices and the last one was an open-question. The reliability of the questionnaire was approved by Cronbach α and it was 0.78. Moreover, by using a panel of experts to review the test specifications and the selection of items, the content validity of this test was improved. The experts were able to review the items and comment on whether the items covered a representative sample of the behavior domain.

Responses to questions 1-9: none, mild, moderate, or severe were given a score of 1, 2, 3 and 4 respectively. After summing the scores, a score of 18 or less was considered as ≪not having sexual problems≫ and a score of 19-27 was considered as ≪having sexual dysfunction≫.

Statistical analysis was carried out using SPSS and Chi-square, Fisher's exact test, and Paired sample t-test were used. A p-value of less than 0.05 was considered to be statistically significant.

## Results

A total of 10 women were excluded from the study. Six of these women had vaginitis and 4 of them had not completed the entire questionnaire.

The data included 40 primiparous women between the ages of 17 and 30. A total of 20 of these women had experienced normal vaginal delivery with mediolateral episiotomy (NVD/epi) and 20 individuals had a caesarean section (C/S) delivery. Among all women, 74.4% were breastfeeding their babies and 46.8% of them had dyspareunia while breastfeeding at 10-15 weeks postpartum. In the NVD/epi group, the majority of individuals were elementary school graduates (55%) compared with 45% middle school graduates in the C/S group. A total of 90% of the women in the NVD/epi group and 75% of the women in the C/S group were housewives and not employed. The age of partners in both groups varied between 20-35 years old. The majority of partners in both groups were middle school graduates and most of them were workers.

It was shown in this study that 55% of the women who had experienced NVD/epi restarted intercourse 40 days after delivery (it varied between 10-90 days) but most of the women in the C/S group (30%) resumed sexual activities and intercourse 10 days postpartum (it varied between 10-60 days). The most common complaint after NVD/epi was decreased libido (80%) compared with 15% prepregnancy. Sexual dissatisfaction (65%), vulvar vestibulitis (60%), vaginal looseness (55%), vaginal dryness (45%), pain upon orgasm (40%), dyspareunia (40%), and vaginismus (40%) were other problems reported after NVD/epi. All of these problems, except vaginal looseness, had existed before pregnancy but they had been considerably less severe [Table 3].

**Table 3:** Women's sexual problems before and after normal vaginal delivery with episiotomy

Sexual problem	Prepregnancy (%)	Postpartum (%)	*P*-value
Vaginal dryness	15	45	0.038
Vulvar vestibulitis	50	60	0.525
Dyspareunia	25	40	0.311
Pain while orgasm	25	40	0.311
Vaginismus	5	40	0.023
Vaginal looseness	0	55	0.001
Postcoital bleeding	0	0	1
Decreased libido	15	80	0.001
Sexual dissatisfaction	15	65	0.001

*P*< 0.05: statically significant

Among women who had a C/S delivery, the most common problem was sexual dissatisfaction (60%) compared with 15% before pregnancy. Decreased libido (35%), vaginal dryness (15%), vulvar vestibulitis (10%), dyspareunia and pain upon orgasm (5%) were the other complaints among individuals in this group.

The noticeable point about post C/S sexual problems was decreasing the severity of vulvar vestibulitis (25% prepregnancy vs. 10% postpartum) and vaginismus (15% prepregnancy vs. 0% postpartum) [Table 4].

**Table 4:** Women's sexual problems before and after caesarean section delivery

Sexual problem	Prepregnancy %	Postpartum %	*P*-value
Vaginal dryness	5	15	0.598
Vulvar vestibulitis	25	10	0.405
Dyspareunia	5	5	1
Pain while orgasm	0	5	0.001
Vaginismus	15	0	0.001
Vaginal looseness	0	0	1
Postcoital bleeding	0	0	1
Decreased libido	5	35	0.048
Sexual dissatisfaction	15	60	0.003

*P* < 0.05: statically significant

## Discussion

We set out to determine how female sexual function is influenced by childbirth. Our study showed that most of the women had some sort of sexual disorder after delivery. The women's responses to sexual stimulants, during early and late postpartum periods, are different and it may offer a frame work for understanding problematic childbearing outcomes.

In our study, all of the women who had a vaginal delivery underwent a mediolateral episiotomy and most of them had more severe sexual problems than others bearing C/S. The women in the C/S group resumed their postpartum sexual activities much sooner than those who had experienced NVD/epi. It is suggested that episiotomy may affect a woman's sexual life during the first year postpartum with more frequent pain, sexual dissatisfaction, and decreased libido. In a cross-sectional study, Byrd,*et al.*(8) found that women who delivered by C/S tend to begin intercourse sooner than average. It seems like the role of an intact perineum is more pivotal than the method of delivery for inducing dyspareunia, whereas it is in contrast with Glazener's(2) findings. Klein,*et al.*(11) showed that both primiparous and multiparous women who had intact perineums after vaginal birth, had less dyspareunia than those undergoing C/S (NVD = 26.2% vs. C/S = 40.7%); however, the proportion of women experiencing dyspareunia was greatest among those who had an episiotomy with or without forceps. In addition, Baksu, *et al.*(12) declared that not only pain but also other important aspects of sexual function, such as arousal, lubrication, orgasm, and satisfaction, are affected by performing a mediolateral episiotomy during vaginal delivery well beyond the puerperal period. Through another study, investigating 110 primipara women, Ejegard(13) showed that women who underwent an episiotomy reported a higher frequency of dyspareunia and insufficient lubrication than women who had given birth without an episiotomy. In similar researches, Solana-Arellano, *et al.,*(14) Williams, *et al.,*(7) and Brown,*et al.*(15) proved the relationship between episiotomies and dyspareunia.

By contrast, the International Randomized Term Breech Trial(16) followed 1596 women and reported that although women who had a C/S birth were less likely to have perineal pain than women experiencing vaginal delivery, the groups reported no differences in rates of dyspareunia (17% of C/S births and 18% of vaginal births). Furthermore, Brown,*et al.*(15) showed that after C/S births or spontaneous vaginal births, women report generally similar rates of morbidity in the long-term.

Presenting contrasting findings, our study supported the hypotheses that considers sexual problems as factors that could impair women's lives and there were statically significant differences between some of those problems before and after pregnancy [Tables 3 and 4].

As opposed to our findings, Clarkson,*et al.,*(17) Xu, *et al.,*(18) and Conolly, *et al.*(19) reported that despite the very common sexual problems after childbirth, sexuality was not significantly associated with delivery type or episiotomy.

In our study, most of the individuals in the NVD/Epi group (30%) and C/S group (60%) had low levels of education. It suggests that not only the pain sensation but also sexual dissatisfaction may occur due to educational and cultural factors. The higher a woman's level of education, the more she will have information about sexual function after giving birth. So, she would recognize how to cope with those following problems and finally her quality of life will be promoted.

We found out that women who breastfed their infants were less interested in starting intercourse than those who bottlefed their babies. Physiologic changes that occur during the postpartum period, such as a decrease in some of the sexual hormones like estrogen and androgens, affect the sexual response cycle. After delivery, the serum level of estrogen is diminished. It can be followed by vaginal dryness and dyspareunia. Diminution of androgens after delivery influences sexual desire and arousal. Hence, sexual activities might be started later after delivery. Glazener, *et al.*(5) and Byrd, *et al.*(8) declared that women who breastfeed tend to begin intercourse later than average. In a similar study, Signorello,*et al.*(20) noted that for all women breastfeeding at 6 months postpartum is associated with a more than four-fold increase in dyspareunia.

Eventually, female sexual dysfunction is a highly prevalent problem for 38-63% of women.(21) It is important because of its disruption of the woman's quality of life. As sexual satisfaction is one variable that may have a positive impact on women's lives.

Although women talk about their sexual problems less than men, it should not be taken for granted. This problem changes a woman's quality of life and can easily disrupt her normal life. The findings of this study support the need for professional nursing attention to human sexuality in achieving the goal of quality postpartum care. That's the duty of clinicians and health care providers to promote the quality of life of postpartum women. There are different approaches to boost women's sexual life. Clinicians should promote their knowledge about sexual function and develop skills on how to talk with women and encourage them to speak about their sexual problems. They can take responsibility for assessing postpartum women's sexual needs with a positive attitude toward sexuality and offering a safe, open, and warm environment for them to express their concerns about sexuality. That is so pivotal; because many women believe that their problems are simply part of having a baby and nothing else. Another important part of work should focus on cultural activities. Despite rapid social changes and influences from western countries, traditional Iranian sexual beliefs persist in most of the regions in Iran. In addition, the meaning of sexuality in Iranian society is based on religious minds, which are primarily patriarchal with women playing submissive and passive roles. Iranian women avoid showing their sexual emotion because of their beliefs and lack of knowledge. So, it seems like the most important step for solving their problems is to elevate their level of information.

It could be done at several times: before pregnancy, through prenatal and antenatal care, and at postpartum visits. In 1998, the World Health Organization(22) recommended that the schedule of postpartum visits should correspond to the times of greatest need for a mother and her infant (i.e., 6 hours, 6 days, 6 weeks, 6 months postpartum). Although the timing of these proposed visits should be flexible to the needs of the mother and the mother should always have easy access to health care. Before pregnancy happens, women and their partners should be aware of potential sexual problems during pregnancy and during the postpartum period. In addition, to maximize the health of postpartum women, obstetricians are expected to both protect the perineum during vaginal birth and avoid unnecessary caesarean birth. After delivery, clinicians can improve postpartum care by talking about sexual intercourse with women and advising them on the use of vaginal lubricants for sexual activity in the first few months after giving birth. This may be prescribed when patients go home after delivery. For breastfeeding women with persistent dyspareunia, a small amount of estrogen cream applied daily to the vagina may be helpful. Health professionals can help patients regain a sense of normalcy after delivery by improving their sexual lives.

Hence, clinicians and researchers are supposed to improve postpartum health care using flexible ways of meeting women's needs and actively listening to their complaints after the birth of a baby. Because the well-being of mothers directly impacts their ability to rear their babies and at last it influences the generations that follow.

### Limitations of this study

Although this study was designed to address the impact of delivery on sexual functioning, maybe because of the limited sample size and sampling method (self-reported questionnaire) we were unable to show a relationship between the method of delivery and all of the postpartum sexual problems. So, it should not be generalized either within this population or across other cultures.

According to the sensitivity of the matter and existence of many conflicts around this subject, further large-scale prospective studies are required to evaluate the impact of successive vaginal deliveries on the long-term risk of sexual problems compared with successive caesarean deliveries.

## Conclusions

Our findings provide some direction for health professionals in assessing postnatal sexual problems. These problems would be able to disrupt a woman's quality of life. Interestingly, sexually satisfying experiences are one component that can contribute to a successful adjustment to the changes associated with childbearing and motherhood. Clinicians, midwives, and all of the health care providers who are engaged in counseling women during the postpartum period should be educated in order to train women regarding potential postpartum problems to help these women promote the quality of their lives.

## References

[1] Cunningham F Gary, Lveno KJ, Bloom SL (2005). Williams obstetrics.

[2] Glazener CM, Abdalla M, Stroud P, Naji S, Templeton A, Russell IT (1995). Postnatal maternal morbidity: Extent, causes, prevention and treatment. Br J Obstet Gynecol.

[3] Thompson JF, Robert CL, Currie M, Ellwood DA (2002). Prevalence and persistence of health problems after childbirth: Association with parity and method of birth. Birth.

[4] Reamy K, White SE (1985). Sexuality in pregnancy and peurperium: A review. Obstet Gynecol Surv.

[5] Glazener CM (1997). Sexual function after childbirth: Women's experiences, persistent morbidity and lack of professional recognition. Br J Gynaecol.

[6] Goetsch MF (1999). Postpartum dyspareunia. An unexplored problem. J Reprod Med.

[7] Williams A, Herronmarx S, Knib R (2007). The prevalence of enduring postnatal perineal morbidity and its relationship to type of birth and birth risk fators. J Clin Nurs.

[8] Byrd JE, Shibley-Hyde J, Delamater J (1998). Sexuality during pregnancy and the year postpartum. J Fam Pract.

[9] MacArthur C, Lewis M, Knox EG (1991). Health after childbirth. Br J Obstet Gynaecol.

[10] Mick JM (2007). Sexuality assessment: 10 strategies for improvement. Clin J Oncol Nurs.

[11] Klein MC, Kazorowski J, Firoz T, Hubinette M, Jorgensen S, Gauthier R (2005). A comparison of urinary and sexual outcomes in women experiencing vagnal and caesarean births. J Obstet Gynaecol.

[12] Baksu B, Davas I, Agar E, Akyol A, Varolan A (2007). The effect of mode of delivery on postpartum sexual functioning in primiparous women. Int Urogynecol Pelvic Floor Dysfunct.

[13] Ejegard H, Ryding EL, Sjogren S (2008). Sexuality after delivery with episiotomy: A long-term follow up. Gynaecol Obstet Invest.

[14] Solana-Arellano E, Villegas-Arrizon A, Legorreta-Soberanis J, Cαrdenas-Turanzas M, Enzaldo de la Cruz J, Andersson N (2008). Women's dyspareunia after childbirth: A case study in a hospital in Acapulco, Mexico. Rev Panam Salud Publica.

[15] Brown S, Lumley J (1998). Maternal health after childbirth: Results of an Australian population based surney. Br J Obstet Gynaecol.

[16] Hannah ME, Hanah WJ, Hodnett ED, Chalmers B, Kung R, Willan A (2000). Planned caesarean vs. planned vaginal delivery for breechpresentation at term: The International Randomized Term Breech Trial. JAMA.

[17] Clarkson J, Newton C, Bick D, Gyte G, Kettle C, Newburn M (2001). Achieving sustainable quality in maternity services-using audit of incontinence and dyspareunia to identify shortfalls in meeting standard. BMC Pregnancy Childbirth.

[18] Xu Xy, Yao Zw, Wang H, Zhou Q, Zhang LW (2003). Women's postpartum sexuality and delivery types. Zhonghua FU Chan Ke Za Zhi.

[19] Conolly A, Thorp J, Pahel L (2005). Effect of pregnancy and childbirth on postpartum sexual function: A longitudinal prospective study. Int Urogynecol J Pelvic Floor Dysfunct.

[20] Signorello L, Harlow B, Chekos A (2001). Postpartum sexual functioning and its relationship to perineal trauma: A retrospective cohort study of primiparous women. Am J Gynecol.

[21] Shokrollahi P, Mirmohammadi M, Mehrabi F, Babaei G (1999). Prevalence of sexual dysfunction in women seeking services at family planning centers in Tehran. J Sex Marital Ther.

[22] World Health Organization (1998). Maternal and Newborn Health / Safe motherhood Unit, Postpartum care of the mother and newborn: A practical guide.

